# A 39 Year mortality study of survivors exposed to sulfur mustard agent: A survival analysis

**DOI:** 10.1016/j.heliyon.2024.e24535

**Published:** 2024-01-17

**Authors:** Hossein Amini, Masoud Solaymani-dodaran, Mostafa Ghanei, Jamileh Abolghasemi, Mahmoud Salesi, Amir Vahedian Azimi, Mohammad Farjami, Amir Hosein Ghazale, Batool Mousavi, Amirhossein Sahebkar

**Affiliations:** a Department of Epidemiology, School of Public Health, Iran University of Medical Sciences, Tehran, Iran; b Minimally Invasive Surgery Research Center, Rasoul Hospital, Iran University of Medical Sciences, Tehran, Iran; c Division of Epidemiology and Public Health, The University of Nottingham, UK; d Chemical Injuries Research Center, Systems Biology and Poisonings Institute, Baqiyatallah University of Medical Sciences, Tehran, Iran; e Department of Biostatistics, School of Public Health, Iran University of Medical Sciences, Tehran, Iran; f Trauma Research Center, Nursing Faculty, Baqiyatallah University of Medical Sciences, Tehran, Iran; g Department of Biostatistics, School of Allied Medical Sciences, Shahid Beheshti University of Medical Sciences, Tehran, Iran; h Student Research Committee, Baqiyatallah University of Medical Sciences, Tehran, Iran; i Prevention Department, Janbazan Medical and Engineering Research Center (JMERC), Tehran, Iran; j Biotechnology Research Center, Pharmaceutical Technology Institute, Mashhad University of Medical Sciences, Mashhad, Iran; k Applied Biomedical Research Center, Mashhad University of Medical Sciences, Mashhad, Iran

**Keywords:** Iran-Iraq war, Sulfur mustard, Generalized gamma distribution, Survival time

## Abstract

**Background:**

The primary objective of this study was to analyze the long-term survival of 48,067 chemical warfare survivors who suffered from pulmonary, cutaneous, and ocular lesions in the decades following the Iran-Iraq war.

**Methods:**

The data for this study were obtained from the Veterans and Martyr Affair Foundation (VMAF) database. The survivors were divided into two groups based on whether they were evacuated/admitted (EA) to a hospital or not evacuated/admitted (NEA) to a hospital. The proportional hazard (PH) assumption for age categories, gender, exposure statuses, and eye severity was not satisfied. Therefore, we used a Generalized Gamma (GG) distribution with an Accelerated Failure Time (AFT) model for analysis.

**Results:**

The study included a total of 48,067 observations, and among them, 4342 (9.03 %) died during the study period. The mean (SD) age of the survivors was 55.99 (7.9) years. The mortality rate increased with age, and higher rates were observed in males. Survival probabilities differed significantly among age categories, provinces, lung severity, and eye severity based on log-rank tests (p-value<0.05 for all). The GG model results showed that higher age and being male were associated with a shorter time to death. The study also found that the mortality rate was significantly higher in the EA group compared to the NEA group.

**Conclusion:**

The present study showed no significant difference in survival time between the EA and NEA groups. The findings suggest that pulmonary lesions caused by mustard gas are more likely to be fatal compared to skin and eye lesions. The results also indicate a potential association between survival time and the severity of lung damage.

## 1 Introduction

Although the exact date of the first synthesis of sulfur mustard gas is unclear, historical reports indicate that it was first synthesized by Despretz in 1822, followed by Niemann and Guthrie in 1860. In 1886, the German chemist Victor Mayer developed the first reliable method for the synthesis of pure sulfur mustard, known as the “Mayer method,” which became the standard procedure for large-scale production of sulfur mustard gas [[1], [2], [3], [4]].

Mustard gas, also known as sulfur mustard, is a highly toxic chemical warfare agent and has earned the nickname “king of war gases” [5]. Exposure to this gas can lead to severe complications in various organs of the body, including ocular and dermal injuries, respiratory tract damage, reproductive and developmental toxicity, and gastrointestinal effects [1,6]. Both acute and delayed effects can occur, with some individuals experiencing late complications even up to 40 years after exposure, as observed in World War I victims [7,8]. Areas exposed to mustard gas remain uninhabitable for extended periods. Temporary vision loss has been reported among mustard gas victims, which has been a significant tactic in numerous military conflicts [9,10]. In 1975, Norman examined the mortality experience of three cohorts of World War I survivors, totaling 7151 white males, including 2718 exposed to mustard gas, compared to 1855 hospitalized with pneumonia and 2578 with extremity wounds [11]. The study revealed that a single exposure to mustard gas resulting in respiratory injury was associated with an increased risk of lung cancer later in life. Following the use of chemical warfare agents in World War I, the UK government commissioned an epidemiological study to address concerns about potential long-term health damage among volunteer ex-servicemen exposed to chemical warfare tests at Porton Down in Wiltshire, UK [12]. The investigation demonstrated that the Porton Down test subjects had significantly higher all-cause mortality compared to non-exposed survivors, with a rate ratio of 1.06. It is worth noting that, apart from warfare and terrorist acts, humans may be exposed to mustard gas in small or large amounts through factors such as factory leaks or activities like fishing in waters where chemical weapons were disposed of [13,14].

The war between Iran and Iraq in the 1980s inflicted lasting physical and psychological damage on the populations of both countries. The use of chemical weapons by the Iraqi army against Iranians, and even their own people, had severe and deadly consequences that persist to this day, even after three to four decades [[15], [16], [17]]. Initially, the use of chemical warfare agents was limited at the start of the Iran-Iraq war. However, over time, despite international agreements to ban chemical weapons [18], the Iraqi army extensively deployed nerve agents like sarin and tabun, as well as mustard gas [19]. Mustard gas was used in more than 387 attacks, involving over 1800 tons of sulfur mustard, targeting both the Iranian military and civilian population [20]. Due to the alkylating mutagenic effects and carcinogenicity of mustard gas, the victims are at risk of developing various types of malignancies later in life, even among those who appeared to have recovered [1,17,21]. In a study by Maynard et al. the fatality rate among World War I soldiers was less than 2 %, whereas it ranged from 3 % to 4 % among Iranian survivors of the Iran-Iraq War [22]. The results also highlighted that the potential for long-term disability resulting from sulfur mustard gas exposure outweighed the fatality rate.

Shirazi and Bilal [23] conducted a comparative study on 77 cases of chemical injury to examine early (one week after exposure) and late (two years after exposure) complications. The results showed that while eye lesions did not show significant changes over time and skin complications tended to decrease, respiratory complications generally worsened.

In another study by Fekri and Johnghorbani [24], skin lesions of 500 survivors exposed to mustard gas were compared with 500 non-exposed survivors. The study revealed a significant association between mustard gas exposure and the occurrence of late skin lesions, including dry skin, hyper- and hypo-pigmentation, regional hair loss, eczema, and chronic urticaria. The prevalence of vitiligo, psoriasis, and discoid lupus erythematosus also increased in the exposed group. Examining the long-term effects of mustard gas on the skin, Begim Mousavi et al. [25] identified various dermal injuries, such as severe itching, dry skin, telangiectasia, melanocytic moles, and cherry angiomas, which persisted for years after the initial injury. Research by Taghdasi et al. [26] focused on long-term respiratory damage in chemical victims of the Kashan city attacks. Mustard gas was found to be the most common chemical agent used, and pulmonary lesions progressively developed in the victims over time. Laluei and Kashanizadeh [27] investigated the outcomes of pregnancy in 50 wives of chemical warfare victims in Kerman province. The study revealed a significant relationship between the severity of the chemical agent effects and low birth weight, abortion, and premature delivery, while no effect on gender outcomes was observed. Khateri et al. [28] conducted a study involving 34,000 Iranians who were studied 13–20 years after exposure to mustard gas. The most common complications observed were lung (42.5 %), eye (39 %), and skin (24.5 %) complications. In a 25-year cohort study by Zafarghandi et al. [29], 7570 Iranian survivors exposed to mustard gas were screened for various cancers, including gastrointestinal, lung, skin, and head and neck cancers. The relative risk of developing malignant disorders was reported as 2.02 for the exposed survivors. Soleimani and Shakirdolagh [30,31] conducted two studies comparing chemical warfare survivors with non-chemical survivors in Sardasht township. The studies revealed significantly worse post-traumatic growth, coping with stress, and higher rates of anxiety or depression among the chemical warfare survivors.

While most studies in this field have focused on investigating the short- and long-term complications of sulfur mustard, this study aims to analyze the survival of 48,067 chemical warfare survivors suffering from pulmonary, cutaneous, and ocular lesions several decades after the end of the Iran-Iraq war. This study represents the first-time survival analyses have been conducted on the entire Iranian sulfur mustard-exposed population.

## 2 Methods

### 2.1 Study group

This was a retrospective cohort study on overall survival of chemical warfare survivors who had been exposed to sulfur mustard during the Iran-Iraq war. All data had been registered in the Veterans and Martyr Affair Foundation (VMAF) database and the analyses were carried out using data up to March 31, 2019. The study was approved by the Ethics Committee Board at the Iran University of Medical Sciences and Janbazan Medical and Engineering Research Center, Tehran, Iran (IR.IUMS.REC.1398.257), which waived the need for obtaining informed consent because this study used nonidentifiable data from an existing data set. The study included both military personnel and civilians who were definitively exposed to sulfur mustard at least once during the Iran-Iraq war (1980–1988). The enrolment of participants was done after obtaining approval from the Veterans and Martyr Affair Foundation (VMAF) of Iran. However, individuals who were chemical warfare survivors (CWS) and met any of the following criteria were excluded from the study: (a) exposure to toxic gases other than mustard gas, and (b) lack of information. Based on the evidence of exposure to SM, the data were categorized into 2 groups [1]: evacuated/admitted (EA), confirmed history of exposure in the affected geographic region with evacuation and hospital admission [2]; not evacuated/admitted (NEA), confirmed history of exposure without evacuation or hospital admission; The severity rankings of injuries to the eyes, skin and lungs were conducted as described recently [32]. In addition, all analyses were performed according to Strengthening the Reporting of Observational Studies in Epidemiology for Respondent-Driven Sampling Studies guidelines [33].

### 2.2 Statistical analysis

All analyses were performed using STATA 15.0 (StataCorp LP; College Station, TX, USA). Data were expressed as frequency (percentages) for categorical variables. The primary outcome was overall survival calculated as the time from the date of SM exposure to the date of death. Patients who remained alive until the last follow-up were censored at that date, and the maximum follow-up time was 39 years. All analyses were completed using data from the VMAF database up to March 31, 2019.

A binary censoring variable was used to indicate whether a patient died of sulfur mustard exposed. Kaplan Meier survival probabilities were calculated and compared between subgroups of predictors using log-rank tests. The mortality rates with 95 % confidence intervals (CI) were also computed. Univariate Cox regressions were fitted to assess the relationships between hazard of death, background and clinical variables to estimate the hazard ratio and 95 % confidence intervals (CIs). The proportional hazard (PH) assumption of this model was assessed by the Schoenfeld residual PH test which was not satisfied for age categories, gender, exposure statuses and eye severity variables.

Different parametric models were compared using Maximum likelihood (ML), Akaike information criteria (AIC) and Bayesian information criteria (BIC) values to evaluate relationships between patient survival and predictors. The generalized gamma model was considered best for this data with the smallest AIC and ML. In addition, the frailty parameter was assessed in this model but was not retained since the frailty parameter was not significant in the model. Therefore, the generalized gamma model was chosen as the best and final model. The Generalized Gamma model is implemented only in the AFT form. The three-parameter generalized gamma survivor and density functions are.

Where is the standard normal cumulative distribution function, and I(a, x) is the incomplete gamma function. A positive coefficient means that time is decelerated by a unit increase in the covariate in question. This may seem awkward, but think of this instead as a unit increase in the covariate causing a delay in failure and thus increasing the expected time until failure. In AFT assumption it places an emphasis on log (time-to failure) rather than risk (hazard) of failure. The generalized gamma distribution works well for most examples with AFT assumption and is used for these data. For AFT models, we can estimate survival time Ratio by exponentiating the coefficient of a variable. For other models, we can calculate the time ratio, TR (p), using appropriate formulae. The times ratio is defined for 0 < p < 1 as the ratio of the corresponding quantile functions, TR (p) = t1(p)/t0(p). The interpretation of TR (p) is that the time required for proportion p of individuals in the exposed or treated population to experience the event of interest is TR(p)-fold the time for the same proportion of events to occur in the reference population [52]. The parameters were estimated by using a maximum likelihood implemented method. p-value <0.05 was considered statistically significant.

## 3 Results

### 3.1 Descriptive

There was a total of 48,067 observations and, of these, 4342 (9.03 %) died during the study period. The total analysis time at risk and under observation was 1,528,288 person-times and the last observed exit time was 39 years. The mean age of survivors was 55.99 ± 7.9 (min-max: 37–98) years. A total of 47,023 (97.8 %) of the subjects were males. The highest number of subjects were distributed in the <51 years-old group. The Documented Exposure Level of 52.6 % of survivors was EA (Table 1).

**Table 1:** Mortality rates per 10,000 persons based on background variables and severity of organ involved.

	Number (%)	Deaths (n)	Person-time	Rate (CI)
**Age category (years)**				
<51	**12,721(26.47)**	538	218,013	24.67(22.67,26.85)
51–53	10,072(20.95)	534	201,540	26.49(24.34,28.84)
53–55	7025(14.62)	413	150,677	27.4(24.89,30.18)
55–60	9272(19.29)	675	228,260	29.57(27.42,31.88)
60+	8977(18.68)	2174	324,118	67.07(64.31,69.95)
**Gender**				
Male	47,023(97.8)	4252	1,098,624	38.7(37.55,39.88)
Female	1044(2.2)	82	0023,984	34.18(27.53,42.45)
**Province**				
West province	18,806(39.1)	1685	439,233	38.36(36.57,40.23)
Non-west province	29,261(60.9)	2649	683,375	38.76(37.31,40.26)
**Documented Exposure Level**				
EA	25,265(52.56)	2504	592,282	42.28(40.65,43.97)
NEA	22,802(47.44)	1830	530,326	34.51(32.96,36.13)
**Lung** severity				
No lesion	22,395(46.59)	2178	520,512	41.84(40.12,43.64)
Mild	20,328(42.29)	1467	469,683	31.23(29.68,32.87)
Moderate	4966(10.33)	605	122,812	49.26(45.49,53.35)
Severe	378(0.79)	84	9601	87.49(70.65,108.35)
**Eye** severity				
No lesion	41,050(85.4)	3776	958,869	39.38(38.14,40.66)
Mild	6719(13.98)	529	156,791	33.74(30.98,36.74)
Moderate	138(0.29)	20	3375	59.26(38.23,91.85)
Severe	160(0.33)	9	3573	25.19(13.11,48.41)
**Skin** severity				
No lesion	42,853(89.15)	3838	999,335	38.41(37.21,39.64)
Mild	4618(9.61)	433	109,047	39.71(36.14,43.63)
Moderate	506(1.05)	54	12,073	44.73(34.26,58.4)
Severe	90(0.19)	9	2153	41.8(21.75,80.34)
**Total** severity				
No lesion	18,158(37.78)	1856	422,352	43.94(41.99,45.99)
Mild	24,039(50.01)	1740	555,401	31.33(29.89,32.84)
Moderate	5279(10.98)	639	130,352	49.02(45.36,52.97)
Severe	591(1.23)	99	14,503	68.26(56.06,83.12)

**Fig. 1:**
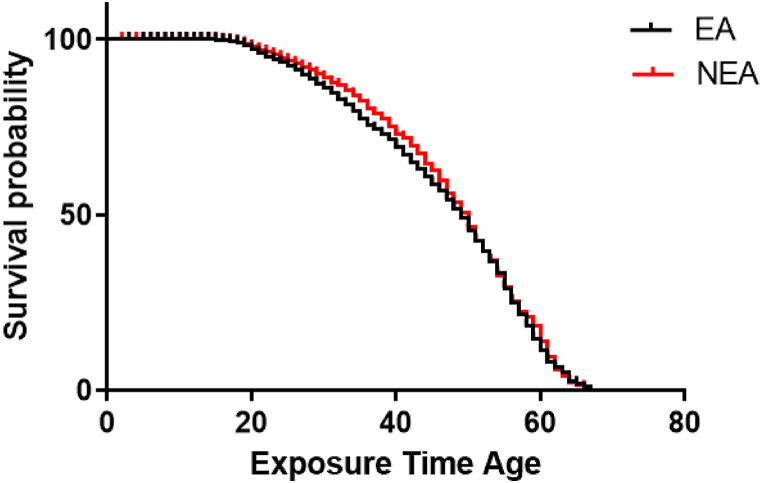
Kaplan-Meier survival probability in Documented Exposure Level.

The mortality rate showed an increasing trend with age, with higher rates in males. This study also found out that mortality rate was high significantly among EA (42.28 deaths per 10,000 populations with CI (40.65,43.97)) than in NEA (34.51 deaths per 10,000 populations with CI (32.96,36.13). The highest mortality rates were observed in the severe ranking of lung severity, followed by the severe total and moderate eye rankings. However, the distribution of mortality was similar across the skin severity categories (Table 1). Kaplan Meier's survival plot is drawn for the EA and NEA groups. According to the graph, it can be seen that the survival of the two groups is the same (Fig. 1).

We used ML, AIC and BIC criteria to evaluate the models and examined the effects of age, gender, province, Documented Exposure Level and severity of lung, skin and eye on survival time using multivariate analysis, with different models. The generalized gamma model chooses as the best means of analysis (Table 2).

**Table 2:** Comparison between parametric regression models with AFT parameterization.

Distribution	ML	df	AIC	BIC
Weibull	−6140.37	18	12316.75	12474.7
Exponential	−13281.1	17	26596.11	26745.29
Log-Logistic	−13281.1	17	26596.11	26745.29
Log-normal	−5667.52	18	11371.03	11528.99
Generalized gamma	−5665.13	19	11368.26	11534.99

ML: Maximum likelihood; df: degrees of freedom; AIC: Akaike information criteria; BIC: Bayesian information criteria; Bold: Lowest value.

The results of the likelihood ratio test for the generalized gamma model indicated a significant contribution of at least one underlying predictor in the model [LR Chi^2^ [47] = 4523.23, p < 0.001]. The results of generalized gamma model showed higher hazard of death for higher age categories, in other words, because the coefficients are positive, the survival time decreases with increasing value, and the hazard of death increases with decreasing survival time (p-value<0.001). Due to the negative coefficient in the gender variable(B = −0.136), the survival time of males is less than females (TR = 0.87), so the hazard of death of males was higher than females (p-value<0.001), but considering that population distribution was unbalanced according to gender in the study, this conclusion needs to be interpreted with caution. The survival ratio is the same for eastern provinces versus non-eastern provinces(p-value = 0.3) as well as for NEA versus EA survivors (p-value = 0.77). This model also showed a lower ratio of death for lung severity in the level of mild and a higher ratio of death in the level of severe versus No lesion (p-value<0.001, Table 3).

**Table 3:** Generalized gamma model with AFT parameterization.

	Coefficient (SE)	TR	95 % CI	P-value
**Age category (years)**				
<51	1			
51–53	0.1(0.006)	1.105	(1.091,1.119)	<0.001
53–55	0.16(0.007)	1.173	(1.157,1.189)	<0.001
55–60	0.281(0.006)	1.324	(1.308,1.34)	<0.001
60+	0.57(0.006)	1.769	(1.748,1.79)	<0.001
**Gender (Male vs Female)**	−0.136(0.017)	0.873	(0.844,0.903)	<0.001
**Province** (Eastern vs Non-Eastern)	0.004(0.004)	1.004	(0.996,1.012)	0.309
**Exposure status** (NEA vs EA)	0.002(0.006)	1.002	(0.991,1.013)	0.772
**Lung severity**				
No lesion	1			
Mild	0.031(0.006)	1.031	(1.019,1.043)	<0.001
Moderate	0(0.007)	1	(0.987,1.013)	0.998
Severe	−0.105(0.017)	0.9	(0.871,0.931)	<0.001
**Eye severity**				
No lesion	1			
Mild	0.019(0.006)	1.019	(1.007,1.031)	0.002
Moderate	0.001(0.033)	1.001	(0.938,1.069)	0.967
Severe	0.048(0.04)	1.05	(0.971,1.135)	0.224
**Skin severity**				
No lesion	1			
Mild	0.002(0.007)	1.002	(0.989,1.016)	0.764
Moderate	0.024(0.02)	1.024	(0.986,1.065)	0.217
Severe	0.022(0.044)	1.022	(0.937,1.115)	0.621

1: Reference category; AFT: accelerated failure time; CI: confidence interval; TR= Time Ratio; SE: Standard Error; CI= Confidence Interval.

## 4 Discussion

The present study observed no significant difference in survival time between the two groups. However, the mortality rate was significantly higher among the exposed group (42.28 deaths per 10,000 population) compared to the non-exposed group (34.51 deaths per 10,000 population). The highest mortality rates were observed in individuals with severe lung severity, followed by those with severe total and moderate eye rankings. However, the distribution of mortality was similar across the different categories of skin severity. Limited studies have reported survival data on the impact of the Iran-Iraq war. In this study, we introduced a novel approach using the GG distribution with an assumed accelerated failure time (AFT). Our contribution advances the field by exploring an area that has not been previously investigated. The Iran-Iraq war, which took place from 1980 to 1988, resulted in numerous deaths and injuries among military personnel and civilians. The extensive use of chemical weapons by the Iraqi army during the war had long-lasting effects on the physical and mental health of thousands of individuals in both countries. Furthermore, it has led to increased mortality rates, persisting even several decades after the ceasefire [34]. The consequences of the war extend beyond the veterans themselves, as their families are also affected [35]. A study examining the immediate effects of mustard gas and nerve agents reported the deaths of 2000 and 3500 individuals, respectively [36].

The EA group is expected to have a shorter survival time compared to the NEA group due to their higher frequency of complications and injuries, resulting in increased mortality. However, it is important to note that chemical survivors in Iran receive free healthcare services, undergo periodic health monitoring, and receive support in terms of social security and pension payments. These services may have contributed to an increased survival time in the EA group, thereby resulting in a similar survival time between the two groups studied.

Our findings also demonstrate the relatively high survival rates among chemical veterans. Sedighe Gharbi et al. [37] highlighted that while the lung lesions in these patients resemble Chronic Obstructive Pulmonary Disease (COPD), there are distinct differences due to varying etiology and clinical care. In a retrospective study examining delayed effects among 1005 chemical warfare victims who died between 1986 and 2003, it was found that 12.1 % of the deaths were attributed to chemical agents [36]. Another study [38] revealed an increased mortality rate attributed to respiratory tract cancers among individuals who worked in a mustard gas factory in the United Kingdom during World War II. Similarly, higher mortality rates due to respiratory cancer were observed in exposed workers at a Japanese mustard gas factory in the years leading up to and during World War II [39]. Mustard gas-associated mortality primarily affected the lungs, accounting for a significant proportion of deaths across mild, moderate, and severe severity ratings. In contrast, a 2017 study conducted in Iran on proportional mortality in the general population reported a potential association between survival time and lung severity in sulfur mustard-exposed patients, particularly in the mild and severe severity categories. These findings complement and expand upon prior knowledge [40]. Our analysis revealed that more than half of the survivors of warfare were in their mid-50s at the time of the study, highlighting the relatively young ages of these individuals during the eight-year duration of the war. This finding is consistent with previous studies [41,42]. Participants from all Iranian provinces were involved in the war efforts [43]. Prior research has demonstrated that exposure to sulfur mustard can lead to the development of a non-invasive method for predicting mortality from high-dose skin exposure [44]. In a study conducted by Gilasi et al. in the Esfahan province, the frequencies of deaths among survivors exposed to mustard gas were slightly higher for neoplasms (30 %) compared to our findings [45]. Furthermore, a retrospective study of 1709 deceased survivors revealed a higher relative frequency of lung disease in exposed individuals (32 %) compared to non-exposed individuals (19 %).

Other studies have reported delayed destructive ocular lesions resulting from mustard gas poisoning, which manifest as itching, burning, and progressive visual deterioration [46]. Additionally, individuals may experience pain, photophobia, decreased vision, ulcerative keratitis, and ultimately blindness [47]. However, neither of these studies demonstrated a direct association between these ocular effects and increased mortality. Collectively, these findings suggest that pulmonary lesions associated with mustard gas exposure have a higher likelihood of being fatal compared to skin and eye lesions. Similar studies conducted in other countries have yielded similar results, supporting the consistency of these findings across different populations.

We found that the proportionate deaths due to mustard gas were relatively low, as described in other studies [[48], [49], [50], [51]]. The univariate Cox proportional hazard model yielded similar results as the log-rank test in evaluating significance of background and clinical symptoms in the analyses. This confirmed that severity of skin lesions did not have a significant influence on survival parametric models. GG and log-normal may be better choices for analysing survival data as these provide analyses associated with survival time without the need for PH assumptions [52]. Due to availability of standard methods such as ML for parameter estimation and testing, and no requirement of PH assumption, AFT models, as parametric models, are attractive [53]. To our knowledge, no other study has considered GG distribution for finding hazard of death or finding prognostic factors in Chemical veterans. In AFT survival models such as GG distribution proportional hazard (PH) assumption is not required. And also these models can specify a direct relationship between the logarithm of survival time and the explanatory variables [54]. However, when PH assumption is met, maybe the result of GG model and Cox models are different. Predictive power of AFT models is higher than those of Cox PH and Cox with time-varying coefficients [55].

In conclusion, the univariate Cox model did not demonstrate significance for certain variables, while the GG parametric model proved to be the most appropriate choice, requiring the inclusion of all variables in the model. In multivariate analysis, the GG distribution provided a better fit to the data compared to other parametric survival models, such as exponential, Weibull, log-normal, and log-logistic distributions. The hazard function in the GG distribution can exhibit a wide variety of shapes [56].

## 5 Limitations and strengths

The limitations of the study included lack of data on the course of progression and rate of late complications, the rate of prompt complications at the time of exposure, the dosage of SM at exposure, smoking habit, the time interval until evacuation and admission to a hospital, the nearness to the assaulted zone and the utilization of protective equipment such as masks. The strengths of the study include the large population size (48,067 chemical warfare survivors). Moreover, the severities of complications in the population were assessed based on expert diagnosis of the VMAF Restorative Commission utilizing guidelines for deciding the rates of chemical harm. Determination of the wounds and seriousness of the injuries within the lungs, skin, and eyes were decided by a board of medical experts.

## Declaration

### Ethics approval and consent to participate

The study was approved by the Ethics Committee Board at the Iran University of Medical Sciences and Janbazan Medical and Engineering Research Center, Tehran, Iran (ID: IR. IUMS.REC.1398.257), which waived the need for obtaining informed consent because this study used nonidentifiable data from an existing data set. The authors declare that the work described has been carried out in accordance with the Declaration of Helsinki of the World Medical Association revised in 2013 for experiments involving humans.

### Data availability statement

The datasets generated and/or analysed during the current study are not publicly available but are available from the corresponding author on a reasonable request.

## Funding

This research did not receive any specific grant from funding agencies in the public, commercial, or not-for-profit sectors.

## CRediT authorship contribution statement

**Hossein Amini:** Writing – original draft, Investigation. **Masoud Solaymani-dodaran:** Writing – review & editing, Investigation, Conceptualization. **Mostafa Ghanei:** Writing – review & editing, Investigation, Conceptualization. **Jamileh Abolghasemi:** Writing – review & editing, Investigation. **Mahmoud Salesi:** Writing – review & editing, Investigation, Conceptualization. **Amir Vahedian Azimi:** Writing – original draft, Investigation. **Mohammad Farjami:** Writing – review & editing, Investigation. **Amir Hosein Ghazale:** Writing – review & editing, Investigation. **Batool Mousavi:** Writing – review & editing, Investigation. **Amirhossein Sahebkar:** Writing – review & editing, Investigation.

## Declaration of competing interest

The authors declare that they have no known competing financial interests or personal relationships that could have appeared to influence the work reported in this paper.
